# Zirconia Implants in Esthetic Areas: 4-Year Follow-Up Evaluation Study

**DOI:** 10.1155/2015/415029

**Published:** 2015-06-01

**Authors:** Andrea Enrico Borgonovo, Rachele Censi, Virna Vavassori, Oscar Arnaboldi, Carlo Maiorana, Dino Re

**Affiliations:** ^1^School of Oral Surgery, University of Milan, Department of Oral Rehabilitation, Istituto Stomatologico Italiano, 20122 Milan, Italy; ^2^Department of Implantology and Periodontology, Istituto Stomatologico Italiano, 20122 Milan, Italy; ^3^Department of Implantology, Dental Clinic, Fondazione IRCCS Cà Granda Ospedale Maggiore Policlinico, School of Oral surgery, University of Milan, 20122 Milan, Italy; ^4^Head Department of Oral Rehabilitation, Istituto Stomatologico Italiano, 20122 Milan, Italy

## Abstract

*Objectives.* The aim is to evaluate the survival and success rates, as well as the marginal bone loss (MBL) and periodontal indexes of zirconia implants positioned in the esthetic jaw areas. *Materials and Method.* 13 patients were selected and 20 one-piece zirconia implants were used for the rehabilitation of single tooth or partially edentulous ridge in the esthetic jaw areas. Six months after surgery and then once a year, a clinical-radiographic evaluation was performed in order to estimate peri-implant tissue health and marginal bone loss. *Results.* The survival and success rates were 100%. The average marginal bone loss from baseline to 48 months after surgery was +2.1 mm. Four years after surgery, the median and the mode for visible Plaque Index and Bleeding On Probing resulted 1 whereas Probing Pocket Depth amounted to 3 mm (SD = ±0.49 mm). *Conclusion.* One-piece zirconia dental implants are characterized by high biocompatibility, low plaque adhesion, and absence of microgap that can be related to the clinical success of these implants even in the esthetic areas.

## 1. Introduction

The original concept of implant surgery as described by Branemark [1] is that the fixture is placed in the bone and completely covered by mucoperiosteal flaps. After the healing period of at least 3 months in the mandible and up to 6 months in the maxilla, the implant is exposed and a healing abutment is connected.

Since the material composition and the surface topography of the implants play a fundamental role in osseointegration, various chemical and physical surface modifications have been developed in order to reduce the time of osseous healing, and it was observed that increased surface roughness of dental implants resulted in greater bone apposition [2] and reduced healing time [3]. However, even if the original protocol by Branemark was modified by modern works of research, patients expect a rehabilitation to be finalized within the shortest time span possible especially if the edentulism involves the esthetic regions. Moreover, patients require implants that are esthetic as well as functional and, for this reason, more recently higher interest is directed towards the esthetic of the prosthetic rehabilitations.

The use of ceramic components based on alumina or yttrium-stabilized zirconium oxide in conjunction with all-ceramic restorations allows to achieve implant osseointegration, which was examined in several animal experiments [4–6], and to solve esthetic problems. In fact, even if several studies reported high success rates for titanium dental implants [7], it is important to consider that bone resorption of the vestibular cortical bone and recession of the peri-implant soft tissue can occur over time [8]. Consequently, the titanium components may be visible and cause discoloration of the gingiva, particularly in cases of thin biotype and high smile line [9]. The first ceramic material that was used in the past for dental implants was aluminium oxide. This material showed good osseointegration but it did not have sufficient mechanical properties for long-term loading [10]. More recently, new generation ceramic materials such as zirconia were introduced. Zirconia is characterized by more favorable mechanical properties (high flexural strength (900–1200 Mpa), hardness (1200 Vickers), and Weibull modulus (10–12)) than aluminium oxide. In addition, this biomaterial has a high biocompatibility and low plaque adhesion [11], and several animal studies showed bone-to-implant contact similar to titanium [5, 6, 12]

The aim of this study is to evaluate the survival and success rates, the marginal bone loss (MBL), radiographic measurements, and periodontal indexes (Plaque Index (PI), Bleeding On Probing (BOP), Probing Pocket Depth (PPD), and implant mobility) of zirconia dental implants positioned in the maxillary and mandibular esthetic areas.

## 2. Materials and Methods

At the Department of Implantology, Dental Clinic, Fondazione IRCCS Cà Granda Policlinico, University of Milan, the authors did a retrospective study of patients treated using monocomponent endosseous zirconia dental implants for the rehabilitation of esthetic areas.

22 one-piece endosseous dental implants made of sintered and yttrium-stabilized zirconium oxide were used for the rehabilitation of single tooth or partially edentulous ridge in the esthetic areas in the maxilla or the mandible. It was considered that the esthetic zone of the jaw includes the central and lateral incisors, the canines, and the first premolars.

The implants used in the clinical study are made of sintered and yttrium-stabilized zirconium oxide (WhiteSky, Bredent, Senden, Germany) and are featured by a conical implant body and a double, cylindrical thread. The endosteal portion has a sandblasted surface, whereas, transmucosally, the implant includes a machined neck with a height of 2 mm. The implant surface is treated with a sanding process. The microscopical surface characteristics of medium rugosity (Ra 0.9-1 m) are similar to the surface of last-generation machine-finished titanium implants.

The abutment surface is smooth and it has a length of 6.8 mm which can be modified by grinding after implant positioning.

**Figure 1:**
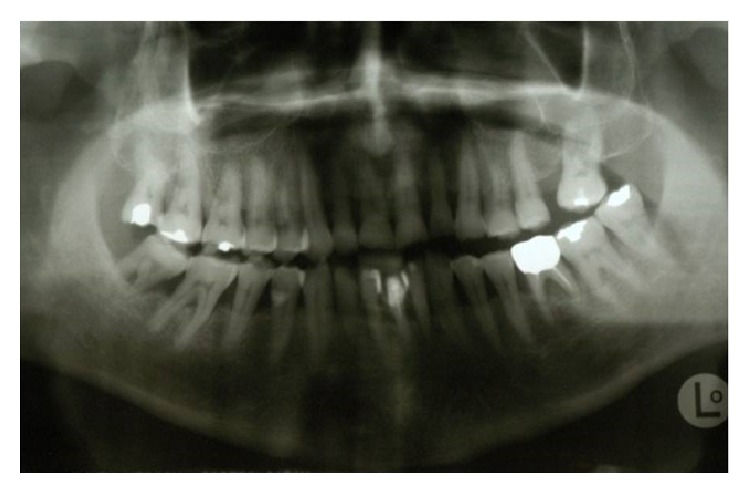
Preoperative orthopantomography.

**Figure 2:**
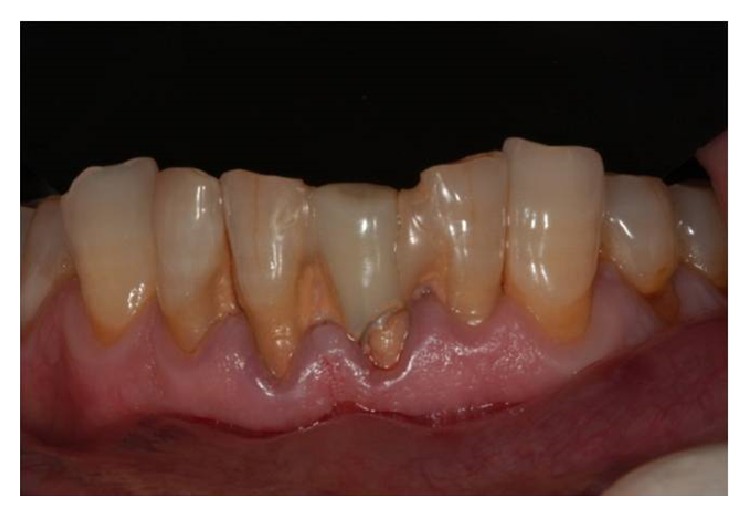
Preoperative clinical view.

**Figure 3:**
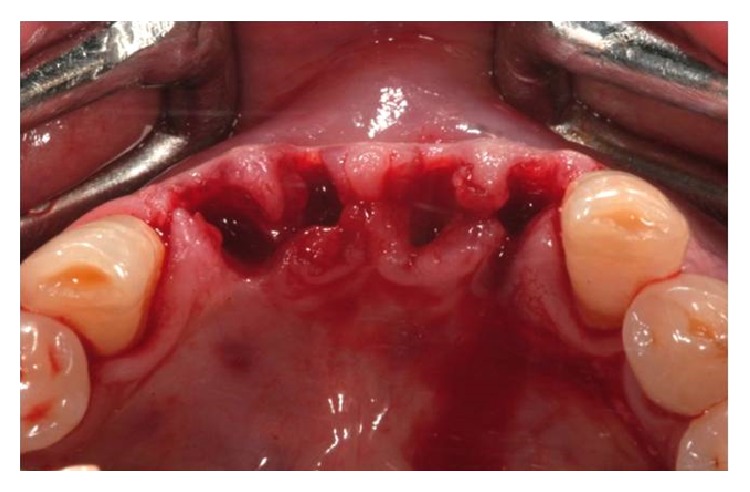
Dental extractions.

For this study, 14 patients in need of a single or multiple teeth replacements in the maxillary esthetic areas were selected (Figures 1, 2, and 3). All sites should present adequate bone volume (minimum bone height and thickness, respectively, of 8 and 5.5 mm). Implants positioned in regenerated bone were excluded from this protocol because the regenerative procedures associated with implant rehabilitation can influence the results in terms of marginal bone loss. In fact, it has been demonstrated that the marginal bone loss is greater in the regenerated bone than in the native bone [13]. Moreover, patients with oral problems such as active periodontal disease or parafunctions, bisphosphonates treatment, smoking more than 10 cigarettes per day, poor oral hygiene, and low compliance and patients with previous or concomitant systemic diseases such as immunodeficiency, head and neck radiotherapy, metabolic disorders, and hematological diseases, together with patients under 18 years of age were not included in this study.

All patients were previously informed about zirconia implants and possible alternatives and gave a written consent. Seven days before surgery, the patients underwent professional oral hygiene and they were instructed to start rinsing mouth twice a day with chlorhexidine 0.2% (Corsodyl, Glaxo, UK) until two weeks after surgery. Antibiotic prophylaxis with 2 gr of Amoxicillin and Clavulanic Acid (Laboratori Eurogenerici, Milan, Italy) was prescribed 1 hour prior to surgery.

**Figure 4:**
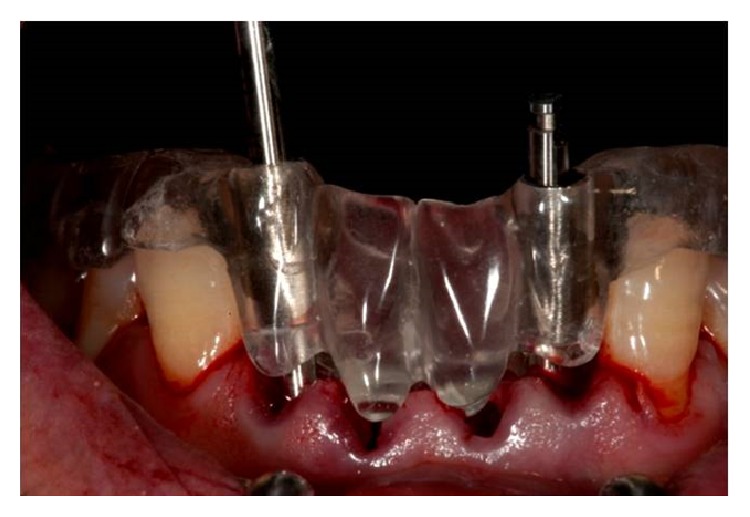
Surgical guide.

**Figure 5:**
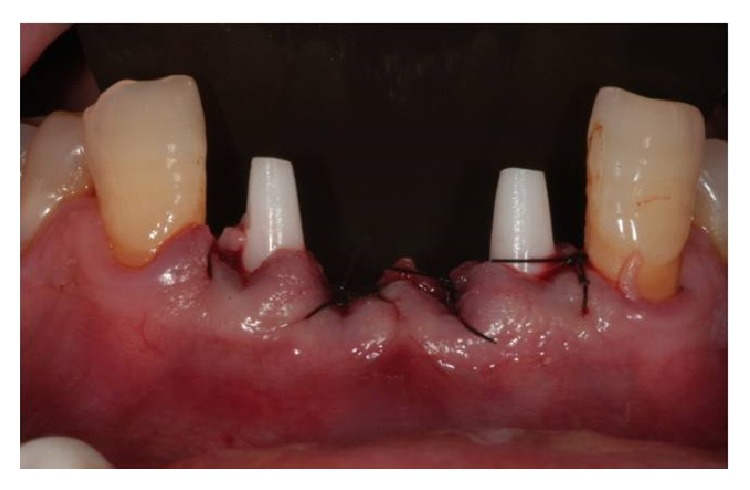
Two monocomponent zirconia implants are placed in the areas 3.2 and 4.2.

The surgical procedure has involved the positioning of implants according to the protocol suggested by Bredent Medical, which is similar to the standard surgical protocol for titanium dental implants. All implants were inserted using a guide device prepared on a diagnostic wax-up (Figure 4). Mucoperiosteal flaps were elevated avoiding vertical releasing incisions in order to reduce the risk of blemishes. After preparing the implant sites, fixture insertion was performed by a surgical microengine. The fixtures were screwed until the rough surface of the implant body was positioned completely inside the bone, whereas implant abutment with smooth neck performed the function of the transmucosal element (Figure 5). All the implants were placed in the correct three-dimensional positioning according to esthetic protocol by Tarnow et al. [14]. Flaps were released through periosteal incisions to attain primary wound closure, and, at the end, flaps were sutured with 4/0 monofilament suture (Premilene, Braun Melsungen, Germany).

After implant insertion, standardized periapical radiography using the Rinn alignment system (Dentsply, Constanz, Germany) with customized silicon bites (Orthogum Zermack, Badia Polesine, Rovigo, Italy) was obtained. The radiographic control was permitted to evaluate the correct positioning of implants.

**Figure 6:**
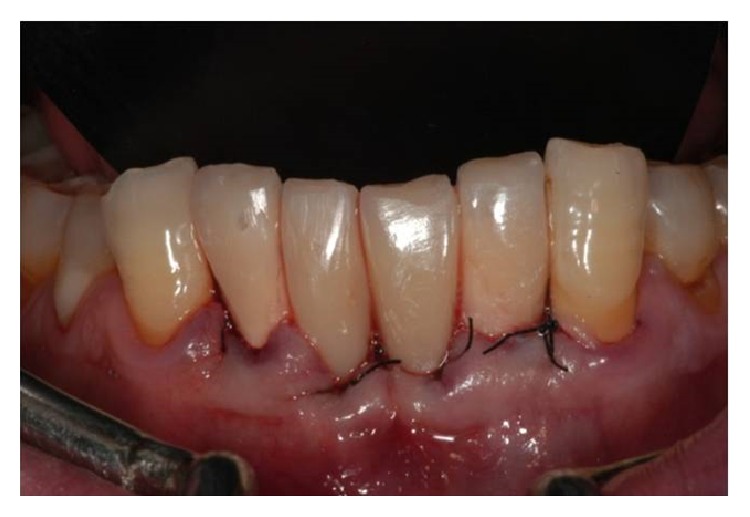
Immediate temporary restoration.

Immediately after surgery, considering that zirconium oxide ceramics are bad thermal conductors, implant abutments were refined in order to correct their axis, length, or undercuts if present, using double diamond burs suited for zirconia (ETERNA Bredent, Senden, Germany) and water cooling. Temporary restorations obtained from diagnostic wax-up were relined with acrylic resin and luted with temporary cement (TEMPBOND, Kerr West Collins Orange, CA, USA). Single restorations were attached to the adjacent teeth by means of composite bonding, whereas multiple implants were connected together by provisional restoration in order to reduce the risk of implant mobility or extra occlusal load (in particular, tongue and lips movements) (Figure 6).

Patients were given oral hygiene suggestions and were instructed not to chew or eat on implant site until healing was completed. Antibiotic therapy (1 gr every 8 hours) and chlorhexidine mouth rinses were continued for 7 days, and Paracetamol 500 mg (Tachipirina, Angelini, Rome, Italy) was prescribed to use if necessity was felt. Sutures were removed 7 days after surgery and follow-up controls were programmed after 1 week, 2 weeks, and, subsequently, once a month for the following 6 months.

**Figure 7:**
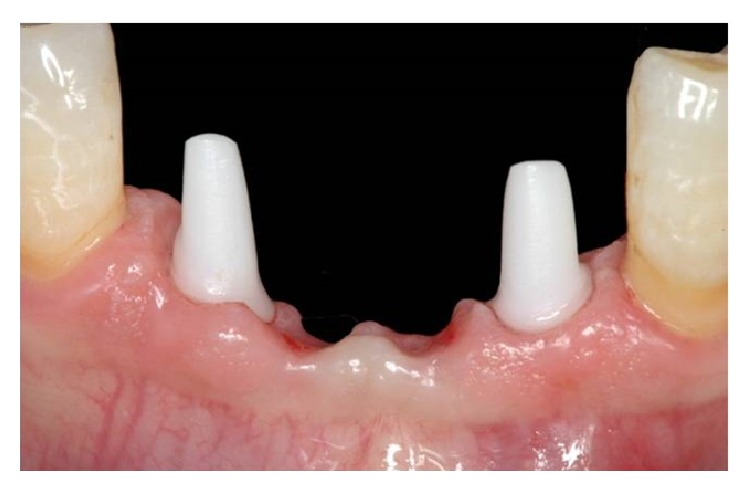
Soft tissue health 6 months after surgery.

**Figure 8:**
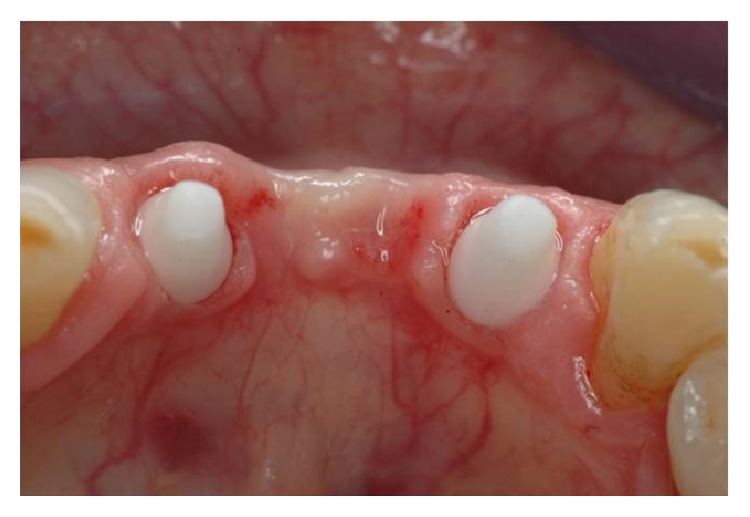
Occlusal view.

**Figure 9:**
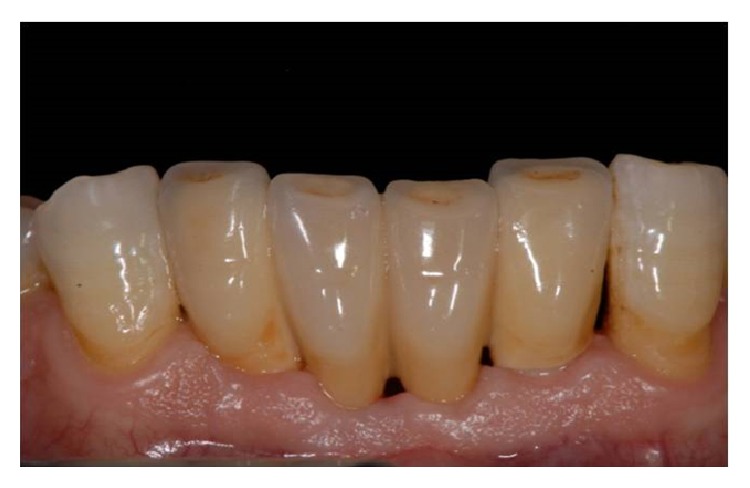
Definitive restoration.

**Figure 10:**
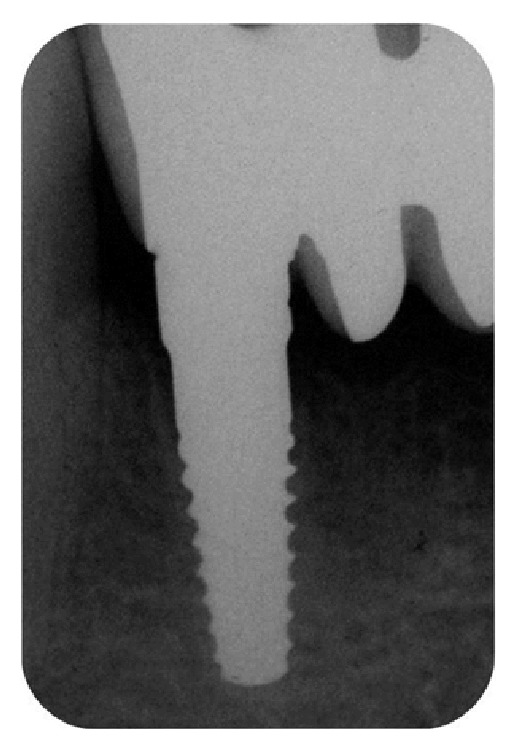
X-ray image of the zirconia implant placed in area 4.2, six months after surgery.

Six months after the surgery (Figures 7 and 8), definitive impressions (IMPREGUM, 3M, ESPE, St Paul, MN, USA) were taken using a retraction cord (Ultrapak Cord, Ultradent, South Jordan, UT, USA) or an impression cap to register implant shoulder margins. The definitive restorations were made with CAD-CAM system (LAVA, 3M, ESPE, St Paul, MN, USA) (Figure 9) and cemented with glass ionomer cement (GC Fuji CEM, GC America, Alsip, IL, USA) (Figure 10).

One week after definitive restorations delivery and, subsequently, every year after implants placement, clinical-radiographic evaluation was performed. The periodontal evaluation was performed using a calibrated probe (Hu-Friedy, N. Rockwell Chicago, IL, USA) and the following periodontal indexes were investigated: Plaque Index (PI), Bleeding On Probing (BOP), Probing Pocket Depth (PPD), and implant mobility.

**Figure 11:**
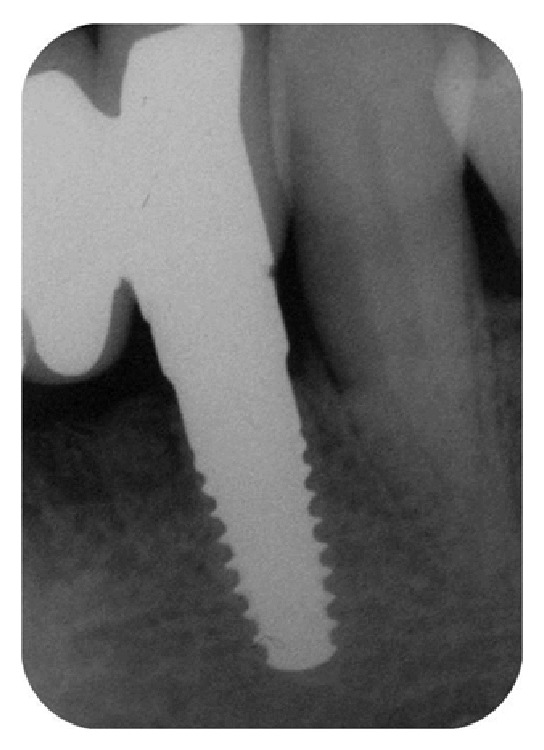
X-ray picture of the zirconia implant placed in area 3.2, six months after surgery.

Moreover, the follow-up protocol included the radiographic control examination (Figures 10 and 11). The radiographs were taken using the customized silicon bite record prepared immediately after surgery. The radiographs were converted in digital images with a scanner (Epson 1680 Pro, Seiko Epson Cooperation, Nagano, Japan) and saved in JPG format. Each image was processed with a specific piece of software (CorelDraw 10.0; Corel Corp and Coral Ltd., Ottawa, Canada) and analyzed at ×20 magnification in order to calculate marginal bone loss. Mesial and distal marginal bone levels of all the implants were measured at baseline and on recall evaluations. The known length of the implant (measured from the implant shoulder to the implant apex) according to the manufacturer was used as a reference point. The distance from implant shoulder to crestal bone level was measured on the magnified images. To analyze the variability, the implant dimension (length) on the magnified X-ray was measured and compared to the real dimension, and ratios were calculated to adjust for distortion. Bone level changes were calculated at the distal and mesial surfaces of all implants by applying the distortion coefficient.

**Figure 12:**
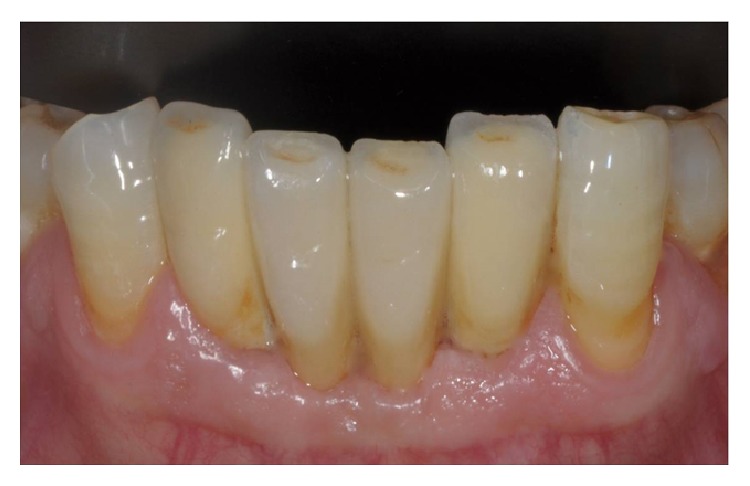
Clinical control 4 years after surgery.

**Figure 13:**
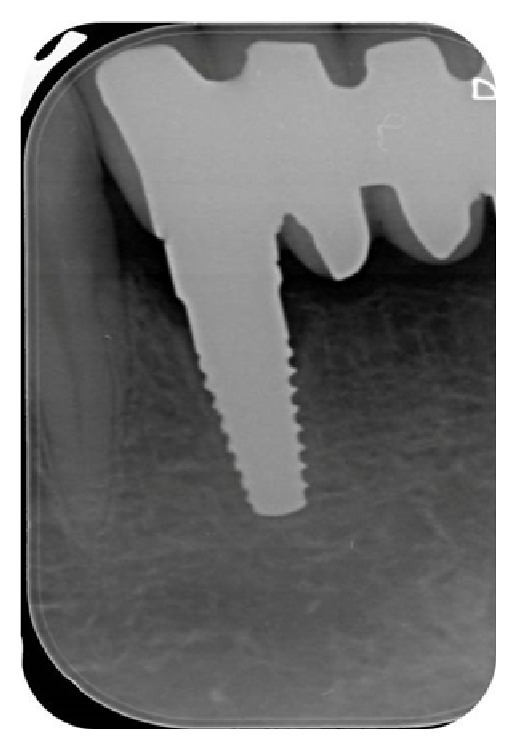
Radiographic control 4 years after implant insertions.

**Figure 14:**
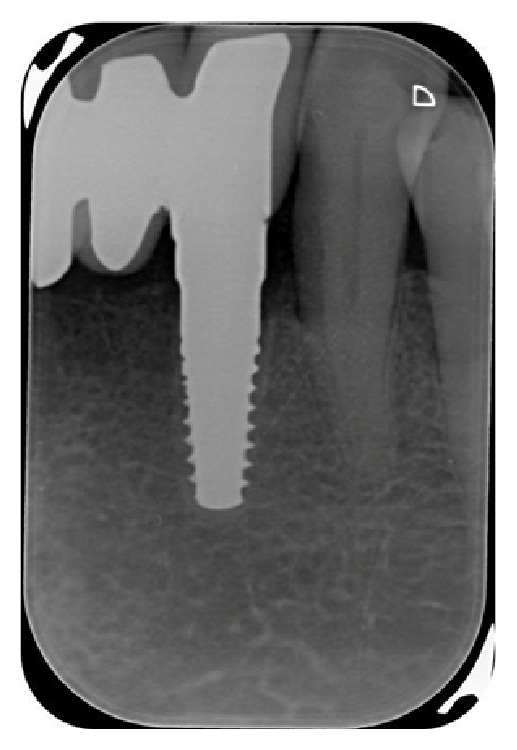
Radiographic control 4 years after implant insertions.

Data analysis was performed with descriptive statistics and the arithmetic mean; the median and the standard deviations were calculated. Clinical and radiographic control examination was repeated every year (Figures 12, 13, and 14).

At the end, success criteria and survival criteria were formulated in accordance with Albrektsson criteria for implants success [15]. Survival criteria were identified as the survival of loaded functionalized asymptomatic implants, whereas success criteria refer to four parameters, absence of implant mobility, absence of self-reported pain or paresthesia, absence of peri-implant radiolucency, and marginal bone loss inferior to 1.5 mm in the first year and to 0.2 mm in the following years.

## 3. Results

At the Department of Implantology, Dental Clinic, IRCCS Fondazione Cà Granda Ospedale Maggiore Policlinico, University of Milan, 14 patients were treated for the rehabilitation of the esthetic jaw areas. Average age was 60 years (ranging from 38 to 75 years), 13 male patients and one female. Starting from January 2007 and recruited in a period of one year, 14 patients were included in the study. The data were recorded to July 2012 when the implants had a minimal observation period of 4 years.

17 implants were placed in the maxilla, whereas 5 implants were placed in the mandible.

Considering the maxillary implants, 10 zirconia dental implants were used for the rehabilitation of single or multiple cases of edentulism in the incisor region; 3 implants replaced the canines, and the other 4 maxillary implants were placed in place of the missing first premolars. All mandibular implants were used for the rehabilitation of edentulism in the incisor region, except 2 implants that were positioned in area of the missing right mandibular first premolar.

Considering patients' selection criteria, one patient with two implants placed in places of the upper right canine and the first premolar was excluded from this protocol because regenerative procedures were performed. For this reason, the data reported in this study refer exclusively to 20 implants. The follow-up period ranged from 6 to 48 months after implant insertion.

During the 48 months of follow-up, no implant failure was reported, with no pain or paresthesia, and, at the radiographic evaluation, peri-implant radiolucency was not detected. Thus, the cumulative survival rate was 100% after 4 years.

At follow-up controls, the median for PI and BOP was 1 and 0, respectively, and the mean values of PI and BOP were 0.54 and 0.23, respectively.

48 months after surgery, the median and the mode for visible Plaque Index (PI) and Bleeding On Probing (BOP) resulted 1. Overall Probing Pocket Depth (PPD) amounted to 3 mm (SD = ±0.49 mm). Mobility was not present at any site, and no pain (spontaneous or on percussion) or paresthesia was reported.

The mean marginal bone level after 4 years was +2,1045 mm, without a difference between mesial and distal sites. In particular, mean marginal bone loss was +1.50 mm (SD = ±1.03) 6 months after implant insertion and +0.446 mm (SD = ±0.64) 6 months after prosthetic finalization.

From 1 year up to 2 years after implant positioning, an improvement of peri-implant bone level value was observed probably due to the formation of new bone trabeculae as a result of maturation of bone (−0.198 ± 0.50 mm).

A minimal bone remodeling with a further marginal bone loss of +0.18 mm (SD = ±0.28) and +0.17 mm (SD = ±0.11), respectively, was also observed at 3 and 4 years follow-up.

For implants placed in the maxilla, the average marginal bone loss from baseline to 6 months after surgery was +1.50 ±1.03 mm, from 6 months to 1 year was +0.65 ± 0.7 mm, from 1 year to 2 years was −0.12 ± 0.57 mm, from 2 years to 3 years was +0.12 ± 0.25 mm, and from 3 years to 4 years was −0.17 ± 0.11 mm.

Four patients were treated for multiple cases of edentulism with 8 zirconia dental implants, and, after surgery, all multiple implants were splinted together by provisional restoration. Considering the marginal bone loss adjacent to free-standing implants and multiple implants, it was observed that there is a statistically significant difference between the two groups (*P* = 0.799).

The success rate was 100%.

## 4. Discussion

The clinical success of the implant rehabilitation is in connection with the interface between bone tissue and implants surface. Several studies showed successful osseointegration of zirconia dental implants in different animal models [5–7, 12]. In the work by Thomsen et al. [16], the interface between the rabbit tibia bone tissue and the surfaces of gold, titanium, and zirconia implants was investigated, and the histological examination disclosed that the bone-implant contact ratio (BIC) is similar for zirconia and titanium implants, whereas gold implants had a lower degree of BIC. In the study by Scarano et al. [17], a great amount of newly formed bones was observed in close contact with the surfaces of zirconia implants positioned in rabbits, and the BIC ratio was 68%. Furthermore, the BIC ratio was better investigated by Akagawa et al. [18] who demonstrated that the bone-implant contact ratio ranged from 54% to 69.8% at 12 months and from 66.2% to 67.7% at 24 months.

The bone-implant contact ratio is the result of bone formation, and it is related to the characteristics of implant surface. Sennerby et al. [19] evaluated the bone tissue reaction to titanium implants and zirconia dental implants with and without different surface modifications. The titanium implants and the zirconia implants with the surface modifications showed the highest surface roughness in comparison to the nonmodified zirconia implants, and, consequently, machined implants presented a lower degree of BIC than titanium and modified zirconia implants.

The reported studies demonstrate a bone-implant contact for zirconia dental implants, similar to those of titanium implants, and these findings suggest that zirconia dental implants can reach firm stability in bones.

More recently, the osseointegration of zirconia dental implants was histologically demonstrated in one human patient [20]. In this study, a two-piece zirconia implant was placed in the maxilla of a healthy woman and 6 months after surgery, the retrieval of the dental implant was performed. The surrounding soft and hard tissues were harvested and processed for histological evaluation. The processed sample of zirconia dental implant provided the histological evidence of osseointegration. Moreover, the scanning electron microscopic analyses showed a good maintenance of the crestal bone level; in fact, it was possible to evaluate that the first bone-to-implant contact was occlusal to the implant-abutment junction.

This finding can be related to the excellent characteristics of zirconia dental implants which present high biocompatibility and low plaque adhesion [17, 21]. In fact, it is important to note that a bacterial adhesion to implant surfaces is the first stage of peri-implant mucositis and peri-implantitis with the resulting loss of the supporting bone in the tissues surrounding the implants [22]. On the contrary, the reduction of bacterial adhesion on the surface of zirconia dental implants promotes early formation of the biological width and, therefore, the formation of a mucosal seal that stops early marginal bone resorption. As demonstrated by Scarano et al. [23], zirconium oxide surfaces showed a significant reduction of the presence of bacteria, and this fact is probably important for the health of the peri-implant soft tissues.

Moreover, the implant system adopted in our study is characterized by monocomponent dental implants. Several studies have shown that bone resorption around the implant neck is related to the presence of the microgap between implant and abutment [14, 24, 25]. This microgap leads to bacterial leakage and a microbial colonization of the gap at the bone level. Peri-implant soft tissues develop an inflammatory response which promotes osteoclast formation and activation to result in alveolar bone loss. According to the authors, the reduction of marginal bone loss is mainly due to the one-piece morphology of zirconia dental implants, through which there is no implant-abutment microgap and its microbial contamination; there are no micromovements of the prosthetic component and repeated screwing and unscrewing [26, 27].

For these reasons, it has been proposed that peri-implant marginal bone loss is more extended around two-piece implants than around one-piece implants as a result of the location of the microgap [28–30].

Another retrospective study suggests that zirconia endosseous implants can achieve a survival rate similar to that of titanium implants with healthy and stable soft and hard tissues. In the work by Brüll et al. [31] 121 zirconia implants (66 two-piece implants and 55 one-piece implants) were inserted in 74 patients. After a mean observation period of 18 months, the cumulative implant survival rate was of 96.5%. The clinical examination revealed that PPD and BOP were statistically significantly lower around implants than around teeth (mean PPD of 1.8 ± 0.4 mm − mean BOP scores of 4.1% ± 4.2%), whereas the radiographic evaluation demonstrated that peri-implant marginal bone levels were stable (mean bone loss of 0.1 ± 0.6 mm) after 3-year follow-up.

Even if the results regarding the rehabilitation of the esthetic areas with zirconia monocomponent implants are encouraging, further scientific information concerning the clinical use of zirconia dental implants is needed, as well as prospective long-term clinical studies in order to understand whether zirconia implants may represent a valid alternative to titanium implants.

## 5. Conclusion

In this study, it was evaluated that there is a preservation of the crestal bone adjacent to zirconia dental implants. In particular, the radiographic measurements of marginal bone loss showed values below 0.9–1.6 mm during the first year in function and not exceeding 0.2 mm 1 year up to 4 years after surgery in accordance with Albrektsson implant success criteria [15]. This finding can be related to some properties which characterize zirconia dental implants. These properties are the high biocompatibility of zirconia surfaces, the low plaque adhesion on zirconia dental implants, and the absence of microgap between fixture and abutment [32, 33].
